# Parotid gland sparing IMRT for head and neck cancer improves xerostomia related quality of life

**DOI:** 10.1186/1748-717X-3-41

**Published:** 2008-12-09

**Authors:** CM van Rij, WD Oughlane-Heemsbergen, AH Ackerstaff, EA Lamers, AJM Balm, CRN Rasch

**Affiliations:** 1 Department of Radiotherapy, Academic Medical Center, Amsterdam, the Netherlands (current address: Erasmusmc, Rotterdam, The Netherlands); 2 Department of Radiation Oncology, The Netherlands Cancer Institute - Antoni van Leeuwenhoek Hospital, Amsterdam, The Netherlands; 3 Department of Head and Neck Oncology and Surgery, The Netherlands Cancer Institute - Antoni van Leeuwenhoek Hospital, Amsterdam, The Netherlands; 4 Department of Radiation Oncology, The Netherlands Cancer Institute - Antoni van Leeuwenhoek Hospital, Amsterdam, The Netherlands; 5 Department of ORL, Academic Medical Center, Amsterdam, the Netherlands, Department of Head and Neck Oncology and Surgery, The Netherlands Cancer Institute - Antoni van Leeuwenhoek Hospital, Amsterdam, The Netherlands

## Abstract

**Background and purpose:**

To assess the impact of intensity modulated radiotherapy (IMRT) versus conventional radiation on late xerostomia and Quality of Life aspects in head and neck cancer patients.

**Patients and nethods:**

Questionnaires on xerostomia in rest and during meals were sent to all patients treated between January 1999 and December 2003 with a T1-4, N0-2 M0 head and neck cancer, with parotid gland sparing IMRT or conventional bilateral neck irradiation to a dose of at least 60 Gy, who were progression free and had no disseminated disease (n = 192). Overall response was 85% (n = 163); 97% in the IMRT group (n = 75) and 77% in the control group (n = 88) the median follow-up was 2.6 years. The prevalence of complaints was compared between the two groups, correcting for all relevant factors at multivariate ordinal regression analysis.

**Results:**

Patients treated with IMRT reported significantly less difficulty transporting and swallowing their food and needed less water for a dry mouth during day, night and meals. They also experienced fewer problems with speech and eating in public. Laryngeal cancer patients in general had fewer complaints than oropharynx cancer patients but both groups benefited from IMRT. Within the IMRT group the xerostomia scores were better for those patients with a mean parotid dose to the "spared" parotid below 26 Gy.

**Conclusion:**

Parotid gland sparing IMRT for head and neck cancer patients improves xerostomia related quality of life compared to conventional radiation both in rest and during meals. Laryngeal cancer patients had fewer complaints but benefited equally compared to oropharyngeal cancer patients from IMRT.

## Introduction

In radiotherapy for head and neck cancer, the major salivary glands frequently receive a high radiation dose. A high dose on the salivary glands results in a reduction of salivary output and a change in its composition [1,2]. This in turn may lead to xerostomia which is cited by patients as a major cause of decreased quality of life (QoL) [1,2]. In recent years Intensity Modulated Radiotherapy (IMRT), has shown to be capable of (partly) sparing the salivary glands and thereby reducing the effect of radiation on these glands [3]. Compared to conventional irradiation, patients receiving IMRT have significantly less permanent quantitative saliva loss after treatment [4-6]. However, the correlation between the amount of saliva and xerostomia related QoL experienced by patients is weak [2,7]. So, the knowledge that IMRT results in a higher quantity of saliva (stimulated or not) does not necessarily mean that patients experience a better xerostomia related QoL.

Does the clinical outcome warrants the extra efforts and costs of IMRT in terms of QoL? Few studies have been performed on xerostomia related QoL in patients receiving IMRT compared to patients treated with bilateral opposed fields [4,8], including small groups of patients. Of these, Jabbari et al reported QoL for a total of forty patients with a minimum follow-up of one year who were treated with standard three-field radiation (n = 10) or matched controlled IMRT (n = 30) [8]. In this study overall QoL scores were better for the IMRT group and improved over time although a statistical significance could not be achieved. Overall, differences in QoL were largest late (> = 6 months) after radiation therapy.

We performed a retrospective analysis of all our patients treated with bilateral radiation and curative intent for head and neck cancer with IMRT or conventional treatment regarding their xerostomia related QoL.

## Patients & methods

### Patients

All patients with T 1-4, N 0-2 M 0 (stage III/IV) head and neck cancer treated with curative intent with bilateral neck irradiation between Jan 1999 – Dec 2003 were selected for this study. Patients who died, had a recurrence or developed a disseminated disease were excluded. The primary tumor (site) received a minimum dose of 60 Gy in 2 Gy daily fractions, 5 fractions a week. For all patients the primary objective was an adequate irradiation of the target volumes. Hundred and ninety-two patients matched these criteria. Since IMRT warrants extra effort, this technique was gradually introduced in our clinic from 1998 onwards. As a consequence not all patients who were eligible for IMRT were treated with this modality. In our study population 77 patients received IMRT; the other 115 patients were treated with bilateral opposed fields and an adjacent supraclavicular field (conventional treatment). The last group (n = 115) was therefore used as control group.

### Questionnaires

During January 2005 all 192 patients were sent a questionnaire on xerostomia (based on the EORTC H&N 35 Questionnaire, Eisbruchs Questionnaire on xerostomia and an additional trial specific questionnaire) [9-12]. Overall response was 85% (n = 163), 97% in the IMRT group (n = 75) and 77% in the control group (n = 88) (Table 1). One of the patients in the control group developed Alzheimer's disease and was excluded from our analysis leaving 87 patients in the control group.

**Table 1:** Patient Characteristics

	IMRT % (n = 75)	Control % (n = 87)
Male (n)	72	64
Female (n)	28	36
		
Mean dose primary tumour	69 Gy	70 Gy
Mean age start RT	59 yrs	59 yrs
Mean interval between RT and Questionnaire	2.3 yrs	2.9 yrs
		
Tumour Site		
Hypopharynx	9	9
Larynx	31	31
Nasopharynx	7	6
Oral cavity	7	8
Oropharynx	37	45
Thyroid	7	0
Other	3	1
		
T-stage		
T1	16	5
T2	37	27
T3	25	33
T4	22	35
		
N-stage		
N0	50	39
N1	15	13
N2	36	48
		
Larynx		
T1-2 (n)	18	15
T3-4 (n)	5	12
		
Oropharynx		
T1-2 (n)	10	5
T3-4 (n)	14	27
		
Concommittant chemotherapy	39	53
Surgery	24	15
Gastrostomy	3	6
Xerostomia related medication	17	20

For the analysis of our data we divided the QoL questions in 2 parts; the first part concerned the questions on xerostomia experienced in rest and the second part questions on xerostomia experienced during meals (Table 2, 3).

**Table 2:** questions related to xerostomia in rest

	Group	Much less %	Less %	Equal %	More %	Much more %	UV *p *value	MV *p *value
Do you have a normal amount of saliva?	IMRT	37	39	18	3	4	0.07	0.008
	Control	58	27	8	2	5		

Has there been a change lately in the saliva amount?	IMRT	7	15	69	8	1	1.0	0.7
	Control	13	14	56	14	3		

		Never %	Sometimes %	Frequent %	Always %			

Is your mouth dry when you are not eating?	IMRT	20	43	20	16		0.004	0.001
	Control	9	30	36	25			

Do you have problems with your gumbs?	IMRT	69	17	8	5		0.3	0.2
	Control	55	32	8	5			

Do you have problems speaking?	IMRT	40	31	24	5		< 0.0001	< 0.0001
	Control	16	28	35	22			

Do you have to drink water during the day because of a dry mouth?	IMRT	20	25	28	27		0.001	0.001
	Control	7	15	35	44			

Do you have trouble sleeping due to a dry mouth?	IMRT	55	25	12	8		0.5	0.2
	Control	48	26	18	7			

Do you have to drink water during the night because of a dry mouth?	IMRT	25	47	19	9		0.05	0.03
	Control	21	32	32	15			

Questions and answers related to xerostomia experienced in rest for IMRT group (n = 75) and control group (n = 87). UV: univariate tested (linear by linear association), MV: multivariate tested (ordinal regression, IMRT tested together with N stage, T stage, prior chemotherapy, prior surgery, interval between RT and questionnaire, current smoker yes/no).

**Table 3:** questions related to xerostomia during meals

	Group	Never %	Sometimes %	Frequent %	Always %	UV *p *value	MV *p *value
Do you have a problem with the transport of solid food through your mouth?	IMRT	32	39	18	11	< 0.001	< 0.001
	Control	12	27	41	21		

Do you have a problem with the transport of grounded food through your mouth?	IMRT	67	19	11	3	< 0.001	0.001
	Control	40	27	24	10		

Do you have a problem with swallowing solid food?	IMRT	32	38	20	10	< 0.001	< 0.001
	Control	15	24	33	28		

Do you have a problem swallowing grounded food?	IMRT	62	19	16	3	0.007	0.02
	Control	42	23	27	9		

Do you experience a dry mouth during meals?	IMRT	39	35	20	5	< 0.001	< 0.001
	Control	14	31	36	19		

Do you need to have a drink of water to swallow your food?	IMRT	23	39	19	19	< 0.001	< 0.001
	Control	6	24	31	40		

Do you find it difficult to eat in front of others?	IMRT	57	24	11	8	0.006	0.02
	Control	35	31	19	15		

		Solid %	Grounded %	Liquid %			

What type of diet do you have?	IMRT	91.	6	3		0.03	0.3
	Control	79	12	10			

		Yes	No				

Do you have to swallow more frequently then you used to?	IMRT	66	34			0.2	0.2
	Control	78	21				

Questions and answers related xerostomia esperienced during meals for IMRT group (n = 75) and control group (n = 87).

UV: univariate tested (linear by linear association), MV: multivariate tested (ordinal regression, IMRT tested together with N stage, T stage, prior chemotherapy, prior surgery, interval between RT and questionnaire, current smoker yes/no)

### Treatment

Irradiation was given on a linear accelerator (4–6 MV Elekta) and all patients were immobilized using custom made masks.

**Figure 1:**
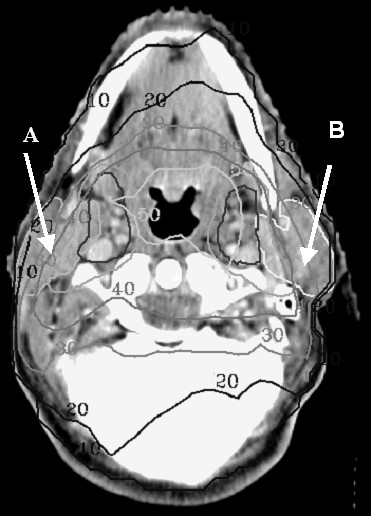
Dose distribution for parotid gland sparing IMRT in Gy, tumor dose 70 Gy. The objective for the parotid gland was set to a mean dose below 26 Gy. A: spared parotid gland, mean dose below 26 Gy, B: sacrified parotid gland, mean dose above 26 Gy.

IMRT was calculated using the University of Michigan planning system (U-M plan, Michigan), 95% of the Planning Target Volume (PTV) had to receive 95% of the prescribed dose. The maximum dose allowed to the spinal cord was 50 Gy. The aim was to reduce the mean dose to 26 Gy or less for at least one parotid gland (Fig. 1). If this was not achievable, the lowest possible mean dose, whilst maintaining target coverage, was accepted. Sparing of the submandibular glands or oral cavity was not attempted. Typically the treatment setup consisted of a five angle coplanar setup and a caudal oblique irradiation field with a total number of segments between 15–20 as described by Eisbruch et al [13]. The control group was irradiated with lateral – opposed photon beams (4 or 6 MV photons Elekta, customized with MLC shielding), after 46 Gy (23 × 2) off-cord reduction was made and the posterior cervical nodes were treated with electrons if necessary. The lower neck nodes were treated using an adjacent anterior photon field. For both treatment groups the same setup correction protocol was used.

### Statistical analysis

For all questions with answers on a three-point or four-point scale (e.g.: never, sometimes, frequently, always), the linear-by-linear association chi-square test was performed to test differences between the control group and the IMRT group (two-sided testing). For questions on a two-point scale, the chi-square test was used. During investigation it appeared that potentially significant factors were not equally balanced over the two groups. In order to make a fair comparison, we corrected for N stage, T stage, chemotherapy, surgery prior to radiotherapy, smoking and length of the interval between start of radiotherapy and answering the questionnaire (interval RT-Q), in a multivariate analysis (MV; ordinal regression). Because the interval RT-Questionnaire was highly correlated with year of treatment this factor was not implemented separately. Since 91% of the patients received the same dose of 70 Gy, 2 Gy per daily fraction, 5 fractions a week, radiation dose was not included as a variable in the MV analysis.

Additionally, we computed an overall score to xerostomia during meals and xerostomia in rest. For this we gave 0 points for all answers related to no xerostomia (e.g. "never a problem") and 1 to a maximum of 3 points for all other answers on the two to four-point scales. We computed the mean overall scores for the total group (IMRT vs. control) and also separately for the largest tumor groups. A higher mean overall score indicated more xerostomia related complaints.

In a multivariate linear regression model we estimated the contribution of all relevant factors to the computed overall scores, using the backward method. This was done for the total score in rest and the total score during meals, separately. Variables tested in this analysis were the same variables as the ones we corrected for in the multinominal logistic regression. In a similar multivariate linear regression model we estimated the contribution of these factors within the IMRT group only, including the contribution of 2 dose parameters (instead of the variable "IMRT yes/no") that were available for these patients. For the statistical analysis we used SPSS software for Windows, release 10.0 (SPSS inc., Chicago, Illinois).

## Results

### Patient characteristics

The two patients groups were not well balanced; most patients had advanced stage head and neck cancer with higher stages in the control group (Table 1). The mean dose to the primary tumor was 69.2 Gy in the IMRT group vs. 69.9 Gy in the control group, the mean interval time between the RT and Questionnaire was 2.3 yrs and 2.9 yrs, respectively. Surgery was performed in 24% of the cases in the IMRT group vs. 15% in the control group. In the surgery-IMRT group one of the submandibular glands was removed in 53% of the cases, both submandibular glands in 12%, 6% had their superficial parotid gland removed another 6% had their superficial parotid gland and one submandibular gland removed, 18% kept all their major salivary glands, in 5% of the patients it was unknown. In the surgery-control group, 60% had one of their submandibular glands removed, 13% had both their submandibular glands removed and 27% had none of their salivary glands removed. Use of medication, which has been recorded as causing salivary dysfunction (antihypertensives, narcotics etc), was equally distributed between both groups. There was a difference between the two groups in the number of patients who received chemotherapy (39% IMRT vs. 53% control group); most patients received platinum based chemotherapy. In the IMRT group, the mean dose to the spared parotid gland was 27.1 Gy (range 15.5 – 60.7 Gy).

### Xerostomia in rest

The mean overall score for 'xerostomia in rest' was 10.3 (4.7 1 SD) for the control group against 7.6 (5.0 1 SD) for the IMRT group (Table 4).

**Table 4:** Mean total scores, Standard Deviation (SD) and Standard Error of the Mean (SEM) of xerostomia during meals and xerostomia in rest questionaires, for IMRT and Control groups.

		Xerostomia during meals			Xerostomia in rest		
	N	Mean	SD	SEM	Mean	SD	SEM

Total Group							

IMRT	75	7.2	5.7	0.7	7.6	5.0	0.6

Control	87	11.5	6.0	0.6	10.3	4.7	0.5

Oropharynx							

IMRT	28	9.1	6.1	1.2	7.0	5.2	1.0

Control	40	13.0	5.5	0.9	10.3	4.3	0.7

Larynx							

IMRT	23	4.3	4.4	0.9	7.1	3.9	0.8

Control	27	9.0	5.9	1.1	10.7	5.0	1.0

Other*							

IMRT	20	7.6	5.4	1.1	8.9	5.7	1.2

Control	24	12.1	6.4	1.4	9.6	5.1	1.1

* hypopharynx, oral cavity, nasopharynx, thyroid

All complaints were reported less frequently in the IMRT group (Table 2), and five out of eight topics scored significantly better in the IMRT group on multivariate analysis (MV). Patients who received IMRT needed to drink water less often during the day and night (p = 0.001 and p = 0.03, respectively). They did not experience a dry mouth as often (p = 0.001) and speaking was less impaired due to a dry mouth (p < 0.001). No statistically significant difference in insomnia complaints was reported due to a dry mouth, although patients in the IMRT group reported to have a normal amount of saliva more frequently than the control group (p = 0.008).

**Figure 2:**
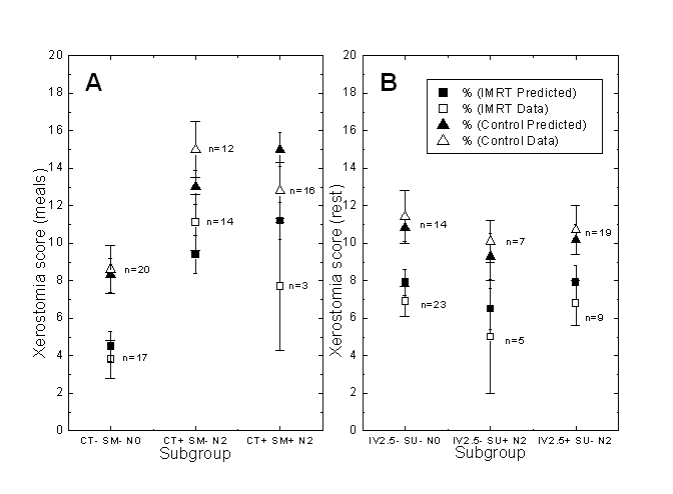
Multivariate backwards linear regression for the largest subgroups, based on the significant predictive factors for xerostomia during meals (Figure 2A) and in rest (Figure 2B). The score (predicted and actual according the data) for IMRT (black square and open square) as well as Control (black triangle and open triangle) for each defined subgroup with standard error are shown. IV2.5-, interval between radiotherapy and questionnaire (RT-Q) of < 2.5 years, IV2.5+: interval RT-Q > 2.5 years, SU-: no previous surgery, SU+: previous surgery, N0: N0 disease, N2: N2 disease. CT-: no chemotherapy, Ct+: chemotherapy.

To see which variables were significantly associated with the overall score, we performed a backward linear regression of the overall score on xerostomia in rest. The factors influencing xerostomia based on the multivariate model (p < 0.1) were: IMRT (p < 0.001), N stage (p = 0.05), interval between RT and questionnaire (p = 0.004) and surgery (p = 0.008). The results indicate that a higher N stage increases xerostomia problems in relation with QoL aspects; in contrast, IMRT, surgery prior to RT and a larger interval between RT and the questionnaire all had a favourable influence on the xerostomia and QoL scores in rest. In Figure 2B the results of the multivariate backwards linear regression are depicted for the largest subgroups, based on the predictive factors for xerostomia in rest. It shows on the left the total scores (predicted and actual) for 23 IMRT patients (■ and □) and 14 control patients (▲ and △) with a relative short RT-Q interval below 2.5 years (IV2.5-), no previous surgery (SU-) and with N0 stage (N0). In the middle the predicted and actual scores for 5 IMRT patients and 7 Control patients also with a relative short RT-Q interval (IV2.5-), previous surgery (SU+) and N2 disease (N2) and on the right predicted and actual scores for 9 IMRT patients and 19 Control patients with a relative long RT-Q interval (IV2.5+), no previous surgery and N2 disease (N2). All scores are shown with the standard error. This Figure shows that the model fits the data quite well and that the IMRT group indeed lowers xerostomia scores in rest within comparable subgroups.

The dose to the parotid glands in the conventional treatment group was not calculated since no planning CT scan was available for all patients. For the patients within the IMRT group a comparison was made between the patients with a mean spared parotid dose above and below 26 Gy. The mean score on xerostomia in rest for the two groups was 8.7 versus 6.5 respectively (p = 0.07).

### Xerostomia during meals

Again all complaints were reported less frequently in the IMRT group (Table 3). The mean total score for xerostomia during meals was 11.5 (6.0 1 SD) for the control group and 7.2 (5.7 1 SD) for the IMRT group (Table 4). Patients who received IMRT reported less difficulty in oral transport of solid and grounded food (both p < 0.001). Also fewer problems with swallowing solid and grounded foods were reported (p < 0,001 and p = 0.02 respectively). A dry mouth was experienced less frequently during meals (p < 0.001), IMRT patients needed to drink water less frequently (p < 0.001) and felt less impaired when eating in public (p = 0.02). The type of diet patients had was not significant at MV analysis. Both groups of patients reported they needed to swallow more often than before radiotherapy.

For the 'xerostomia during meals score' we found the following relevant factors on backward linear regression (with a MV p value < 0.1): IMRT (p < 0.0001), N stage (p = 0.002), chemotherapy (p = 0.08) and current smoking (p = 0.06)). IMRT lowers the overall score while the presence of the other factors increases the score.

In Figure 2A the results of the multivariate backwards linear regression are also depicted for the largest subgroups, based on the predictive factors for xerostomia during meals. It shows on the left the total scores (predicted and actual) for 17 IMRT patients (■ and □) and 20 Control patients (▲ and △) without chemotherapy (CT-), no current smoking (SM-) and N0 disease (N0). In the middle the predicted and actual scores for 14 IMRT patients and 12 Control patients with chemotherapy (CT+), also no current smoking (SM-) and N2 disease (N2) and on the right predicted and actual scores for (only) 3 IMRT patients and 16 Control patients with chemotherapy (CT+), current smoking (SM+) and N2 disease (N2). The data show that the model fits the data quite well, except with regard to the effect of smoking, comparing the CT+, SM-, N2 group with the CT+, SM+, N2 group: the model predicts higher scores for both the IMRT and Control group in the latter group because of the smoking whereas in fact the actual data show for both IMRT and Control group a lower score.

For the patients within the IMRT group a comparison was made between the patients with a mean spared parotid dose above and below 26 Gy. The mean score on xerostomia during meals for the two groups was 8.3 versus 5.2 respectively (p = 0.014).

#### Artificial saliva

We tried to analyze whether there was a difference between the groups in the use of artificial saliva (none, sometimes, often, and always). Only 13 patients reported using artificial saliva (7 with regularity), which limited a reliable analysis of this subject. Distribution of the 13 patients was 10 in the control group (6 sometimes, 2 often, 2 always) against 3 in the IMRT group (sometimes) (linear by linear association: p = 0.04).

#### Tumor groups

The effect of IMRT on overall xerostomia scores within the different tumor groups was also analyzed. The largest tumor subgroups in this study are the larynx and the oropharynx (Table 4). With regard to xerostomia during meals, the number of complaints is higher in the oropharynx group and lower in the larynx group, when compared to the total group (9.1, 4.3 and 7.2 for IMRT and 13.0, 9.0 and 11.5 for oropharynx, larynx and control, respectively). The relative difference between IMRT and control group is quite similar for all subgroups. It shows a lower overall score of about 35–45% compared to the score in the control group. None of the two specified tumor sites (larynx, oropharynx) was a significant factor at the performed backwards regression on the overall score. For the score on xerostomia in rest, the level of complaints is similar for all subgroups (range 7.0–7.6 for IMRT, 10.3–10.7 for control). For the IMRT group, the dose to the oral cavity and parotid gland was 58 vs 25 Gy and 26 vs 24 Gy for the oropharynx and larynx patients respectively. The score of the patients receiving an IMRT technique improved in time (correlation coefficient of dose and time 0.36 (p = 0.002) and 0.34 (p = 0.004) for the parotid gland and oral cavity dose respectively).

#### Including dose parameters for the IMRT group

For most patients of the IMRT group (N = 71) we had additional dose data available concerning the dose at the organs at risk (mean dose to the spared parotid gland, mean dose to the oral cavity). The mean volume of the oral cavity was 108 cm^3 ^(1 SD 21 cm^3^) and the mean volume of 1 parotid gland was 23 cm^3 ^(1 SD 8 cm^3^). The mean dose was 43 Gy and 28 Gy, respectively. We repeated the linear regression for the scores of "xerostomia during meals" and "xerostomia in rest" within the IMRT group only, adding the 2 dose factors to the model. Again chemotherapy and smoking remained in the model for "xerostomia during meals", surgery and the interval RT-Q for "xerostomia in rest" (N stage does not remain in the model now for both endpoints). Furthermore, the mean dose to the spared parotid gland was significantly associated with both endpoints at MV analysis whereas the mean dose to the oral cavity was not. When oral cavity was tested alone (UV), it was a significant predictor (p = 0.007) for xerostomia during meals but not for xerostomia in rest (p = 0.5). The univariate p-values for the mean dose to the spared parotid gland were 0.001 and 0.01 for "xerostomia during meals" and "in rest" respectively. With respect to the individual items on the questionnaire, both dose parameters showed the strongest correlation with the same items: oral transport and swallowing of solid food and with the item of a dry mouth when eating: all three a correlation coefficient of 0.4 with the mean dose to the spared parotid gland (p < 0.01) and of 0.2–0.3 with the mean dose to the oral cavity (p < 0.05).

## Discussion

Our results showed that patients receiving IMRT had a better xerostomia related QoL than patients who received bilateral opposed radiation fields. Other studies either were non-significant or dealt with IMRT patients alone, however, the results in our study were in line with these publications [4,14]. The conventionally treated en IMRT treated patient groups were not well balanced. By means of correcting for significant factors found in ordinal regression multivariate analysis we were able to correct for discrepancies between the two patient groups.

Frequently, a difference was made between xerostomia in rest and xerostomia during meals. The parotid glands were said to be largely responsible for the saliva output during meals whereas the oral cavity and submandibular glands are supposed to be mainly responsible for lubrication in rest [15]. For this reason xerostomia in rest and xerostomia during meals were used as endpoints in our analysis.

The aim of our treatment was to spare (one of) the parotid glands i.e. reducing the mean parotid dose to below 26 Gy. Sparing of the submandibular glands and oral cavity was not an objective since this could not be achieved together with irradiation of level II on both sides. However, our results not only showed a marked difference in experience of xerostomia during meals, but also a difference in xerostomia experienced in rest. Within the IMRT group the distinction between the patients with a mean spared parotid dose below and above 26 Gy pointed in the same direction. The total score for xerostomia during meals was significantly better for the below 26 Gy group. On multivariate analysis the dose to the parotid gland was a significant contributing factor as the dose to the oral cavity was not. For the xerostomia in rest a similar trend was found within the IMRT only group but this trend was not significant. Earlier reports on QoL after salivary gland sparing IMRT except for Jabbari et al made no distinction in QoL during meals and during rest. Eating QoL was reported by Jabbari et al to be better in the IMRT group; however the difference was not significant [8]. In general: the differences between the conventional and the IMRT group emerged largest and most significant by the xerostomia during meals questions. Within the IMRT group the mean dose to the spared parotid gland correlated most with the xerostomia during meals score (Pearson correlation 0.4, p = 0.001) and less with the xerostomia in rest score (Pearson correlation 0.3, p = 0.014) confirming that although parotid gland sparing IMRT improves QoL compared to conventional radiation for both topics, the largest effect is still on xerostomia during meals.

Swallowing difficulties are not caused by xerostomia alone. Eisbruch et al. reported that damage to the pharyngeal constrictors may cause dysphagia and aspiration in patients receiving intensive chemotherapy and radiotherapy [16]. This effect is considered to be independent of the irradiated volume [14]. Whether a swallowing organs sparing IMRT technique is effective is as yet unknown.

IMRT, pre-radiotherapy surgery and the time interval between therapy and answering the questionnaire all had a positive effect on the overall xerostomia during rest complaints. As for IMRT and a longer time interval after radiation, the results are in line with other publications describing long-term recovery of xerostomia [3,4,8]. The effect of pre-radiation surgery was a new finding. A possible reason for this could be that the primary tumor region was treated to a lower dose: 60–66 (12/32 39% patients in the surgery group, compared to 1% in the non-surgery group) instead of 70 Gy. However, for the surgery-IMRT group, the dose to the parotid gland and oral cavity was not reduced (data not shown). The type of surgery was equally distributed between the two groups.

The larynx cancer group had a better mean xerostomia during meals related QoL than the other patients (Table 4). Although the laryngeal xerostomia score with conventional fields was similar to the scores in oropharyngeal patients with IMRT the estimated absolute benefit from IMRT was the same. Direct comparison between Oropharynx and laryngeal cancer patients should be done with care. Despite the retrospective nature of the analysis the results imply that for oropharyngeal patients there is still progress to be made. In earlier reports on xerostomia QoL only few laryngeal patients were included, which made a comparison between tumor groups impossible. Regarding the xerostomia in rest the difference between the larynx patients and the other patients was non-existing (Table 4).

Although the current study was not prospective and our IMRT group and control group were not entirely balanced on multiple issues (T stage, N stage, time from treatment to questionnaire, concomitant chemotherapy), we were able to correct this statistically and the differences in QoL scores remained significant at ordinal regression analysis.

## Conclusion

Compared to conventionally irradiated head and neck cancer patients, IMRT treated patients had improved xerostomia related QoL during meals and in rest. Even though in this retrospective study oropharyngeal cancer patients had fewer complaints than laryngeal cancer patients; IMRT improved xerostomia related QoL for all reported tumor sites, including the larynx. Within the IMRT group the xerostomia scores were better for those patients with a mean parotid gland dose to the "spared" parotid gland below 26 Gy.

## Competing interests

The authors declare that they have no competing interests.

## Authors' contributions

CvR gathered data and was the main author of the manuscript. WO performed statistical analysis. AA advised in the Quality of Life questionaires. EL gathered treatment planning data. AB revised the manuscript and aided in the analysis. CR was the senior author and major contributor to the manuscript and analysis.
